# Morphological analysis of tumor microenvironment in HER2-positive breast cancer: predicting response to neoadjuvant chemotherapy on histopathological images

**DOI:** 10.1186/s13058-025-02139-x

**Published:** 2025-10-21

**Authors:** Wensheng Cui, Ming Fan, Lihua Li

**Affiliations:** 1 School of Computer Science and Technology, Hangzhou Dianzi University, Hangzhou, 310018 China; 2 Institute of Biomedical Engineering and Instrumentation, Hangzhou Dianzi University, Hangzhou, 310018 China; 3 School of Computer Science and Information Technology, Daqing Normal University, Daqing, 163712 China

**Keywords:** Breast cancer, Deep learning, Histopathological images, Neoadjuvant chemotherapy, Tumor microenvironment

## Abstract

**Background:**

HER2-positive breast cancer (HER2 + BC) is clinically distinct from other subtypes, such as triple-negative or hormone receptor–positive breast cancers, due to its unique tumor microenvironment (TME) and its heterogeneous response to neoadjuvant chemotherapy (NAC). Given the critical role of the TME in treatment outcomes, we investigated whether TME features extracted from histopathological images can predict pathological complete response (pCR) and guide personalized therapy.

**Methods:**

We retrospectively analyzed 147 HER2 + BC patients treated with NAC, including 85 from the Yale Response dataset (training cohort) and 62 from the IMPRESS HER2+ dataset (external validation cohort). Hematoxylin and eosin-stained histopathology images were segmented using VGG-16 and Xception networks to generate tissue segmentation images (TS-images). Based on the TS-images, tumor and stroma regions were segmented. Intratumoral and stromal tumor-infiltrating lymphocytes (iTILs and sTILs, respectively) were extracted from these regions and then combined to form TILs. The morphological features of these regions were quantitatively characterized using connected component analysis. Feature selection was performed by integrating morphological and clinical data via the least absolute shrinkage and selection operator. The selected features were then used to train a multilayer perceptron model, which was validated on the IMPRESS HER2+ dataset.

**Results:**

In external validation, the model based on sTILs achieved an AUC of 0.873 for pCR prediction, with an F1 score of 0.889, PPV of 0.821, recall of 0.970, and NPV of 0.933. This performance substantially outperformed models trained on stroma (AUC = 0.779), tumor (0.732), iTILs (0.594), and TILs (0.668). Notably, the sTILs-based model maintained high performance (AUC = 0.722) even when trained with 20% of the training cohort. Univariate analyses identified morphological predictors for pCR, including the filled area of significant regions (mean) in sTILs (*P* value = 0.015).

**Conclusion:**

Morphological TME features from histopathological images can accurately predict pCR in HER2 + BC, supporting their use in guiding NAC decision-making.

**Supplementary Information:**

The online version contains supplementary material available at 10.1186/s13058-025-02139-x.

## Background

HER2-positive breast cancer (HER2 + BC) represents a biologically and clinically distinct subtype of breast cancer, accounting for approximately 20–25% of all cases [1, 2]. Compared to HER2-negative breast cancers, HER2 + BC is associated with more aggressive behavior and a higher risk of recurrence [3–6]. Unlike hormone receptor–positive (HR+) tumors, which tend to be indolent and endocrine-responsive, or triple-negative breast cancer (TNBC), which is immunologically active yet lacks effective targeted therapies, HER2 + BC presents a complex tumor biology shaped by HER2 overexpression, high proliferation, genomic instability, and variable immune infiltration [7, 8]. The development of HER2-targeted therapies has dramatically improved outcomes, yet approximately 30% of patients fail to achieve pathological complete response (pCR) following neoadjuvant chemotherapy (NAC) combined with anti-HER2 treatment [9–12]. This highlights the urgent need for predictive biomarkers that can stratify patients prior to treatment and guide clinical decision-making. Notably, the tumor microenvironment (TME) of HER2 + BC exhibits substantial heterogeneity in immune and stromal composition, differing markedly from that of TNBC or HR+ subtypes. These distinctions position HER2 + BC as a biologically relevant context for studying TME-related predictors of therapeutic response.

At present, conventional clinical indicators, including tumor size [13], pathological grade [14], Ki-67 proliferation index [15], and tumor-infiltrating lymphocytes (TILs) [16–18], have limited accuracy in predicting pCR. To overcome this limitation, researchers have proposed molecular biomarker-based methods, such as CALGB 40601 [19], SPAG5 [20], and PD-L1 [21]. However, these approaches often involve considerable costs and time-consuming procedures. In addition to clinical and molecular indicators, artificial intelligence (AI) has increasingly been applied to the analysis of medical images for predicting responses to NAC. Most of these efforts have focused on radiological images, particularly magnetic resonance imaging [22, 23] and positron emission tomography/computed tomography [24]. Compared with radiological images, histopathological images, which remain the standard for diagnostic evaluation, provide more detailed information about the TME and offer valuable insights into disease progression [25, 26].

Researchers have applied AI to characterize TME information from immunohistochemistry (IHC)-stained breast cancer histopathological images [27, 28]. Although non-routine IHC staining methods, such as Ki-67 and PHH3, can capture high-value biomarkers within the TME and improve the predictive accuracy of pCR, they also pose challenges, including higher medical costs, longer assay times, and greater reliance on manual processes. As a result, an increasing number of studies have focused on using routinely stained histopathological images, such as hematoxylin and eosin (H&E), to identify TME-related biomarkers. For instance, Fisher et al. extracted TME information from triple-negative breast cancer histopathology images and found that the interaction between tumor cells and TILs had significant predictive value for pCR [29]. Shen et al. analyzed histopathological images using AI to extract nuclear features, which were then used in machine learning (ML) models to predict responses to NAC [30]. Li et al. proposed the tumor-associated stroma score as a means of forecasting NAC response in breast cancer patients [31]. These studies suggest that the morphological characteristics of tissues within the TME are associated with treatment responses to NAC in breast cancer [32, 33]. Nevertheless, despite these advances, there remains considerable potential for further exploration of the TME through histopathological image analysis.

In this study, we applied deep learning (DL) techniques to segment H&E-stained core needle biopsy histopathological images of HER2 + BC. This segmentation process produced tissue segmentation images (TS-images). Based on these TS-images, we extracted morphological features from different tissue components of the TME, including tumor, stroma, intratumoral TILs (iTILs), stromal TILs (sTILs), and TILs, in order to predict pCR to NAC. The primary objective of this research is to assist clinicians in patient selection, enhance NAC treatment response rates, and contribute to the advancement of precision oncology.

## Materials and methods

### Datasets

A total of 147 patients who received NAC at Yale University and Purdue University were retrospectively included in this study. Model development was performed using the Yale Response dataset, while external validation was conducted using the IMPRESS HER2+ dataset to assess generalizability. Clinical and histopathological characteristics of the patients are summarized in Table 1. To compare characteristics between the pCR and non-pCR groups, categorical variables (e.g., estrogen receptor (ER) / progesterone receptor (PR) status) were analyzed using the Chi-square test, while ordinal variables (e.g., Nottingham grade and Nuclear grade) were compared using Fisher’s exact test. Continuous variables were assessed using the Mann–Whitney U test.

**Table 1:** Clinical and histopathological characteristics of HER2+ patients in the two datasets

Cohort	Characteristics	Case#/median	%/Range	*P* value
Yale Response dataset	Total case number	85	–	–
	Cases with pCR	36	42.35%	–
	Cases with residual tumor	49	57.65%	–
	ER-positive (ER+)	69	81.18%	0.878
	PR-positive (PR+)	66	77.65%	0.773
	HER2/CEP17 ratio	3.14	0.00–17.40	**0.027**
	Residual tumor size (cm)	1.35	0.02–11.00	**< 0.001**
	HER2 copy number (HER2CN) (signals/cell)	11.0	3.3–6.7	0.136
IMPRESS HER2+ dataset	Total case number	62	–	–
	Cases with pCR	38	61.29%	–
	Cases with residual tumor	24	38.71%	–
	ER+	30	48.39%	**0.042**
	PR+	19	30.65%	**0.019**
	HER2/CEP17 ratio	6.73	1.23–22.98	** < 0.001**
	Residual tumor size (cm)	0.80	0.10–7.00	** < 0.001**
	Residual cancer burden	1.39	0.91–4.14	** < 0.001**
	Age (years)	56	30–76	0.104
	Nottingham grade	1	1.61%	1.000
	Ⅰ			
	Ⅱ	27	43.55%	
	Ⅲ	34	54.84%	
	Nuclear grade	0	0.00%	1.000
	Ⅰ			
	Ⅱ	10	16.13%	
	Ⅲ	52	83.87%	

*P* values indicate statistical significance (*P* value < 0.05)

The Yale Response dataset consisted of whole slide images (WSIs) from pretreatment core needle biopsies, along with corresponding clinical information from 85 female patients diagnosed with HER2 + BC [34]. All WSIs were scanned at 20 × magnification using the Aperio ScanScope Console (version 10.2.0.2352). Before surgery, each patient received trastuzumab either alone or in combination with pertuzumab. The efficacy of NAC was evaluated by board-certified pathologists based on surgical resection specimens. The definition of pCR followed institutional standards and referred to the absence of residual invasive carcinoma, lymphovascular invasion, or metastatic disease. Among the 85 patients, 36 (42.35%) achieved pCR, while the remaining 49 (57.65%) were categorized as non-pCR.

The IMPRESS HER2+ dataset included 62 patients with HER2 + BC, each represented by H&E-stained WSIs of pretreatment core needle biopsies and associated clinical data [27]. Slides were digitized at 20× magnification using a Hamamatsu scanner. Most patients received NAC regimens that included Taxol (paclitaxel or docetaxel) in combination with trastuzumab. A subgroup of seven patients was treated with a four-cycle regimen comprising pertuzumab, trastuzumab, and docetaxel. Among these, four patients achieved pCR and three did not. The definition of pCR in this cohort was consistent with that used for the Yale dataset. In total, 24 patients (38.71%) achieved pCR, while 38 (61.29%) were classified as non-pCR.

### Overview of the framework

**Fig. 1:**
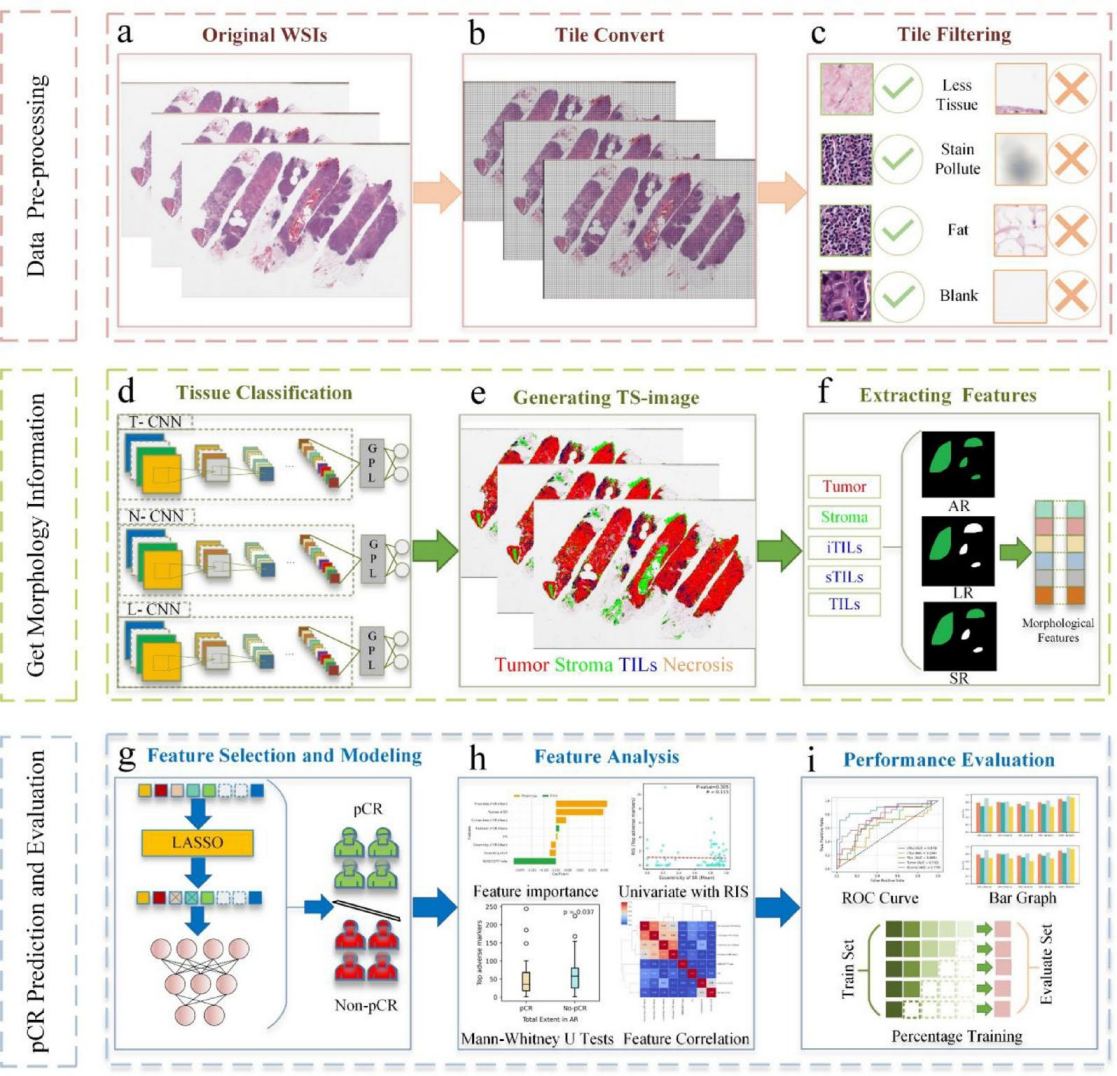
Overview of our pipeline. **a**–**c** illustrate the preprocessing steps, including the conversion of WSIs into tiles and filtering non-informative tiles. **d**–**f** depict the classification of multiple tissue types, the generation of TS-images, and the extraction of morphological features from tumor, stroma, iTILs, sTILs, and TILs. g–i present the construction of the pCR prediction model, the analysis of selected features, and the evaluation of model performance. T-CNN is designed for tumor classification, N-CNN identifies necrotic regions, and L-CNN is applied to classify lymphocyte infiltration, GPL, global maximum pooling layer; TILs, tumor infiltrating lymphocytes; iTILs, intratumoral TILs; sTILs, stromal TILs; AR, all regions; SR, significant regions; LR, largest regions

Our methodology consists of three main components, including histopathological image pre-processing, TS-image generation with morphological information extraction, and the modeling approach for pCR prediction and evaluation. Figure 1 illustrates this comprehensive pipeline.

### Histopathological image pre-processing

Digital pathology images typically comprise billions of pixels, making direct input into DL models computationally infeasible [35–37]. To overcome this limitation, we employed the OpenSlides toolkit to tile whole-slide images (WSIs) into smaller tiles of 175 × 175 × 3 pixels [38]. WSIs frequently contain ink artifacts, blank areas, sparse tissue regions, and fat, which may introduce irrelevant noise. To mitigate this, Otsu’s thresholding method was applied to identify tissue-containing regions and quantify the tissue pixel proportion in each tile. Tiles with a tissue pixel fraction below 20% were excluded from further analysis to ensure that only research-relevant regions were retained.

### Tissue classification and generating tissue segmentation image

Three transfer learning models were implemented using TensorFlow, each trained to classify tile-level regions into tumor, necrosis, or lymphocytes compartments. WSIs were reconstructed to generate TS-images that segment tumor, stromal regions, and TILs, including iTILs and sTILs. These TS-images enable systematic feature extraction, enhance model generalizability, and provide contextual insights beyond conventional pixel-level analysis.

In this study, we conducted image-based analyses to characterize the TME based on spatially distinct tissue compartments that are biologically representative and observable in H&E-stained core needle biopsy slides. Specifically, the TME was delineated into five compartments, comprising tumor, stroma, iTILs, sTILs, and overall TILs, thereby reflecting both the structural and immunological components of the tumor-associated microenvironment. Non-informative regions such as adipose tissue, necrotic zones, ink artifacts, and blank areas were excluded during preprocessing. Tissue segmentation was performed on WSIs using DL models trained on pathologist-annotated regions, ensuring reproducible delineation of TME compartments in accordance with current consensus standards. The stromal region refers to the supportive tissue within the TME, comprising extracellular matrix, fibroblasts, vascular structures, and immune cells that do not infiltrate tumor nests. sTILs are lymphocytes located within stromal areas, including peritumoral stroma and inflammatory zones, while iTILs are lymphocytes that directly infiltrate tumor. This compartmental definition enables precise attribution of morphological features to biologically distinct regions within the TME, thereby enhancing their interpretability and relevance for pCR prediction.

**Fig. 2:**
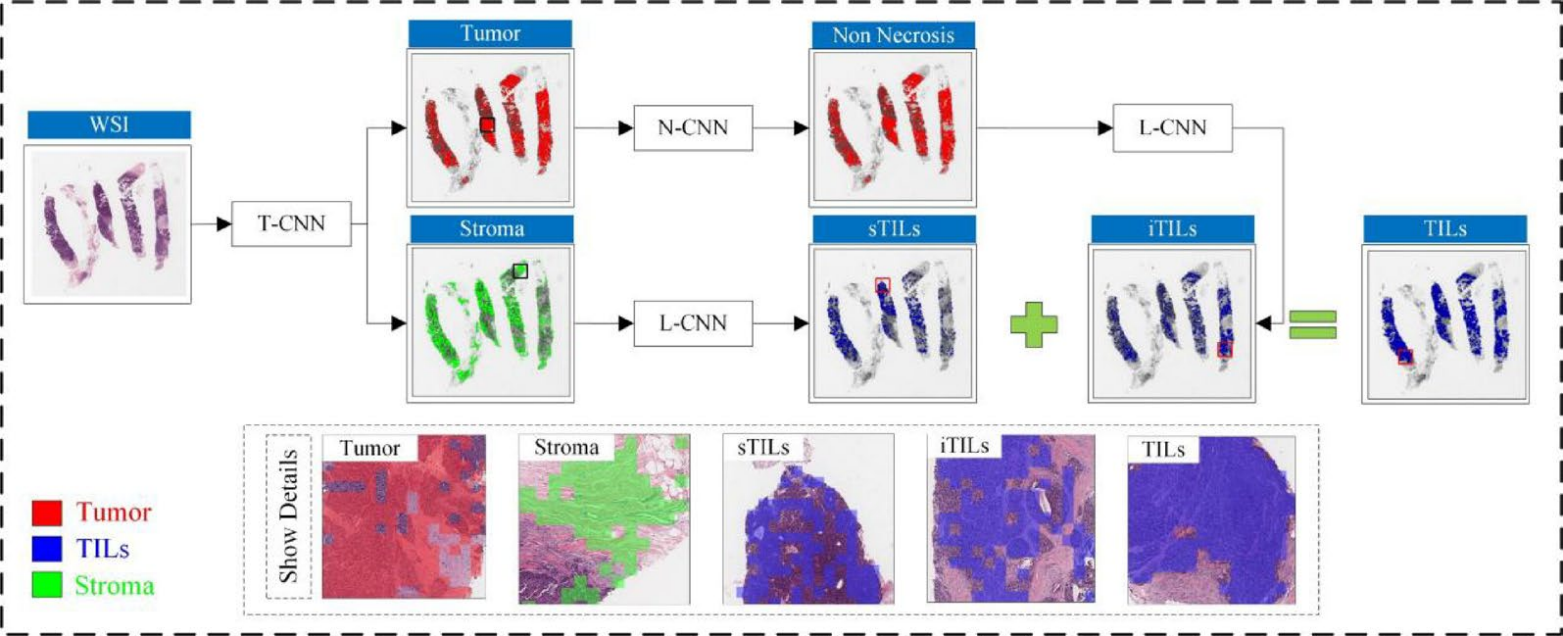
Workflow for histopathological region classification using whole-slide images from the IMPRESS HER2+ dataset (patient ID: 080_HE) T-CNN is designed for tumor classification, N-CNN identifies necrotic regions, and L-CNN is applied to classify lymphocyte infiltration

Due to sample size disparities across tissue categories, a sequential binary classification strategy was adopted instead of a multi-task framework. As shown in Fig. 2, the pipeline first distinguishes tumor from stromal regions. A dedicated convolutional neural network (CNN) is then used to detect necrosis, given its morphological similarity to iTILs [39]. Subsequently, iTILs and sTILs are identified within the respective tumor and stromal regions. These subsets are aggregated to define the complete TILs compartment used for downstream analysis. As the primary objective of this study centers on morphological characterization rather than classification methodology, additional technical details are provided in Additional File 2.

### Feature extraction and construction

We utilized the publicly available Scikit-image library’s regionprops module to extract morphological features from TS-images, encompassing tumor, stroma, sTILs, iTILs, and TILs. Each tile was treated as the unit of analysis, enabling a whole-slide evaluation of tissue morphology. To preserve structural continuity, eight-connectivity was applied to identify connected components within each tissue type supporting comprehensive morphological assessment across WSIs. Using Scikit-image functions, we extracted 46 morphological features per tissue region, following approaches similar to Diao et al. [40] and Wang et al. [41]. These features capture diverse tissue characteristics, including size-related metrics (e.g., component count, major and minor axis lengths) and shape descriptors (e.g., Euler number, eccentricity).

To comprehensively characterize the spatial properties of the TME, we adopted three distinct feature extraction strategies. The all-region method analyzes all connected tissue components, providing a global view of tissue architecture. The largest-region method focuses on the single largest component within each tissue type, representing the dominant structural pattern. The significant-region method evaluates components exceeding 5% of the largest area, using the mean and standard deviation of their features to capture local morphological variation and TME heterogeneity.

**Fig. 3:**
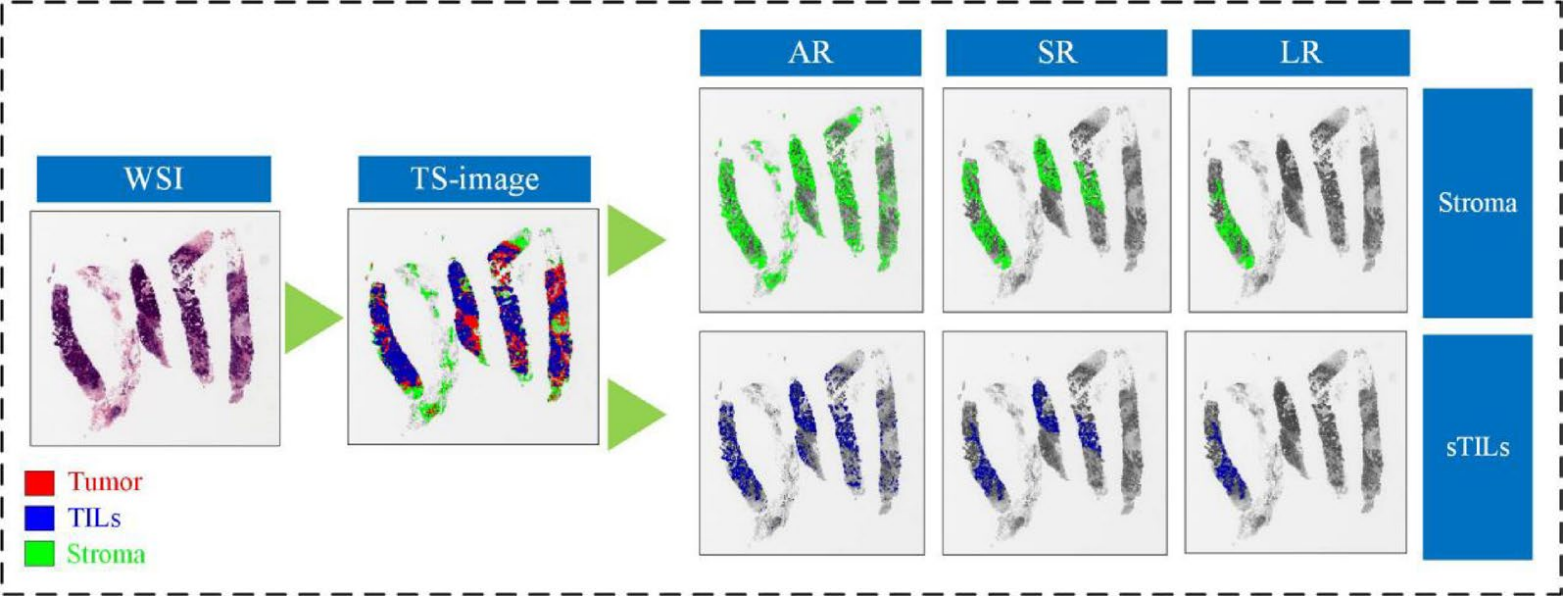
Three strategies for feature extraction from stromal and sTILs regions in the IMPRESS HER2+ dataset (patient ID: 080_HE). AR, all regions; SR, significant regions; LR, largest regions

The selection of the percentage threshold for significant regions is critical for extracting biologically relevant morphological features. Overly high thresholds may miss key structural patterns, whereas low thresholds can introduce noise and impair model generalizability. Details of the threshold determination process are provided in the experimental section. For each tissue region, we extracted 11 feature sets from the largest-region, 12 feature sets from the all-region, and 23 feature sets from the significant-region. The feature names and their corresponding descriptions are provided in Additional File 1. Figure 3 depicts the three perspectives used for extracting morphological features from stromal and sTILs regions.

In addition to morphological information, we integrated clinical features into our analysis. Studies have demonstrated that clinical variables such as ER/PR status and the ratio of HER2 gene copy number to chromosome 17 centromere signals (HER2/CEP17 ratio) are significantly associated with patient prognosis [42–44]. Incorporating these features was intended to enhance predictive performance and support a more comprehensive evaluation of treatment response. Clinical data were merged from the Yale Response and IMPRESS HER2 + cohorts. Specifically, we included ER status and percentage, PR status and percentage, and the HER2/CEP17 ratio [45]. All categorical clinical variables (e.g., ER, PR) were numerically encoded prior to modeling. Specifically, binary variables were mapped to 0 and 1 (e.g., ER-negative = 0, ER-positive = 1), ensuring compatibility with ML algorithms, including MLP.

### Machine learning framework for predicting pCR to NAC

Data preprocessing began with feature standardization, scaling all input variables to have a mean of zero and a standard deviation of one. To reduce redundancy and improve generalization, we applied the least absolute shrinkage and selection operator (LASSO) for feature selection, with the regularization parameter optimized via stratified tenfold cross-validated grid search. The selected features were subsequently used to train a multilayer perceptron (MLP) classifier. To mitigate potential overfitting due to the limited sample size and high-dimensional feature space, L2 regularization (weight decay) was incorporated into the MLP, and its hyperparameters were independently tuned using the same cross-validation strategy. Model performance was evaluated on an independent external validation cohort.

### Statistical analysis methodology

We conducted analyses of morphological and clinical features from the Yale Response and IMPRESS HER2+ datasets using the Mann–Whitney U test for continuous variables. Categorical variables were analyzed using the Chi-square test or Fisher’s exact test, depending on the distribution of counts across categories. Furthermore, we utilized Spearman's rank correlation coefficients to evaluate the relationships between morphological features and residual infiltration size (RIS), as well as the interrelationships among morphological features. These coefficients yield a correlation measure (R) and a corresponding two-sided *P* value. For all statistical analyses, we employed a *P* value threshold of less than 0.05 to indicate statistical significance.

### Model performance evaluation metrics

We employed various metrics to evaluate model performance, including area under the curve (AUC), F1 score, positive predictive value (PPV), recall, and negative predictive value (NPV). In the validation results from independent multicenter datasets, an AUC above 0.800 was considered indicative of excellent model performance, whereas an AUC above 0.700 reflected moderate discriminative capability.

### Experimental environment

The hardware configuration for all experiments comprised a high-performance computing cluster equipped with two NVIDIA Quadro RTX 6000 GPUs for parallel computing and a 2.0 TB hard disk for storage. For software, the OpenSlide toolkit (version 1.2.0) was employed for WSI tiling, while TensorFlow (version 2.4.1) was utilized for data loading, deep model training, and testing. Python was used in combination with the Scikit-learn (version 1.2.2) and SciPy (version 1.8.1) libraries to perform ML and statistical analysis. In addition, OpenCV (version 4.7.0), Pandas (version 2.0.2), and Scikit-image (version 0.19.3) libraries were used for image morphology feature extraction and data processing, respectively.

## Results

### Evaluation of significant region thresholds

The appropriate selection of the significant region threshold is crucial for accurately capturing the spatial characteristics of the TME. To systematically evaluate its impact, we tested thresholds of 1%, 3%, 5%, and 10% by extracting morphological features under each setting. Threshold selection was conducted through tenfold cross-validation on the training set (Yale Response dataset) using both morphological features alone and their combination with clinical variables. As detailed in Additional File 2 (Table S1 and Table S2), the 5% threshold achieved the best overall predictive performance and was therefore selected for subsequent analysis. This threshold was then applied to the external validation cohort, where its generalizability was supported by consistent predictive accuracy. Morphological and clinical features were further integrated to predict pCR in HER2 + BC patients receiving NAC. Results on the external validation cohort (IMPRESS HER2+ dataset) are presented in Table 2, with morphology-only performance reported in Additional File 2 (Table S3).

**Table 2:** Comparison of the generalization performance of morphological and clinical feature combinations for five tissue types under different significant region thresholds in the IMPRESS HER2+ dataset

Region	Sign. Thresholds (%)	AUC	F1 score	PPV	recall	NPV
Tumor	1	0.716	0.756	0.705	0.816	0.600
	3	0.708	0.764	0.667	**0.895**	0.615
	5	**0.732**	0.740	0.771	0.711	0.586
	10	0.724	**0.805**	**0.795**	0.816	**0.680**
Stroma	1	0.717	0.701	0.692	0.711	0.520
	3	0.726	0.795	0.775	0.816	0.667
	5	**0.779**	**0.883**	**0.745**	**0.946**	**0.846**
	10	0.734	0.806	0.714	0.926	0.727
iTILs	1	**0.684**	**0.780**	**0.727**	**0.842**	**0.650**
	3	0.651	0.714	0.694	0.735	0.583
	5	0.594	0.654	0.708	0.607	0.571
	10	0.620	0.667	0.667	0.667	0.588
sTILs	1	0.774	0.805	0.775	0.838	0.683
	3	0.822	0.827	0.756	0.912	0.778
	5	**0.873**	**0.889**	**0.821**	**0.970**	**0.933**
	10	0.719	0.684	0.650	0.722	0.700
TILs	1	0.675	0.709	0.683	0.737	0.522
	3	**0.691**	0.698	**0.759**	0.647	0.581
	5	0.668	**0.730**	0.676	**0.793**	0.632
	10	0.676	0.688	0.647	0.733	**0.667**

Best-performed values are highlighted in boldface

Sign., significance; TILs, tumor-infiltrating lymphocytes; iTILs, intratumoral TILs; sTILs, stromal TILs; PPV, positive predictive value; NPV, negative predictive value

Table 2 compares model performance across five tissue types under varying thresholds. The 5% threshold consistently yielded superior performance, particularly for stroma and sTILs. In the stroma, it achieved the highest AUC (0.779), F1 score (0.883), and NPV (0.846), demonstrating its effectiveness in capturing spatial features relevant to pCR while minimizing irrelevant tissue interference. For sTILs, the 5% threshold yielded the best AUC (0.873), F1 score (0.889), PPV (0.821), recall (0.970), and NPV (0.933), highlighting its ability to preserve key TME characteristics. In contrast, the 1% threshold resulted in the best recall and AUC for iTILs, but at the cost of poorer performance in other regions, such as stroma and sTILs. This suggests that its inclusion of more marginal regions may be beneficial in certain contexts (e.g., iTILs) but introduces noise elsewhere. The 10% threshold, while producing the highest F1 scores in tumor and stroma, underperformed in iTILs and sTILs, indicating potential over-simplification and loss of spatial specificity. Although the 3% threshold showed competitive performance, it did not consistently outperform the 5% setting. Therefore, the 5% threshold was identified as optimal, balancing noise reduction and feature retention to enhance model generalizability.

### Machine learning utilizing morphological and clinical features to predict the pCR

We employed CNNs to segment histopathological images into five distinct regions, namely tumor, stroma, sTILs, iTILs, and TILs. For each region, 46 morphological features were extracted. In addition, five patient-level clinical features (HER2/CEP17 ratio, ER, PR, ER percent, and PR percent) were included. Feature selection was performed using LASSO, and the selected features were input into a MLP to predict pCR in HER2 + BC. To justify the selection of MLP as the primary classifier, we conducted additional benchmarking against three classical ML models, namely Logistic Regression, Support Vector Machine, and Random Forest. All classifiers were trained on the same LASSO-selected feature sets to ensure comparability. As reported in Additional File 2 (Tables S4 and S5), the MLP consistently achieved superior predictive performance, particularly in critical regions such as stroma and sTILs, where it yielded the highest AUC and F1 score. These results suggest that, even with a relatively small dataset, the MLP effectively captured complex nonlinear relationships between clinical and morphological features, supporting its use as the core predictive model in this study. Table 3 presents model performance on the external validation set along with comparisons to baseline and published models.

**Table 3:** Generalization performance validation of pCR predictions in the IMPRESS HER2+ dataset

Type	Method	AUC	F1 score	PPV	recall	NPV
Mor. + Clinical	Tumor	0.732	0.740	0.771	0.711	0.586
	Stroma	0.779	0.883	0.745	0.946	0.846
	sTILs	**0.873**	**0.889**	0.821	**0.970**	**0.933**
	iTILs	0.594	0.654	0.708	0.607	0.571
	TILs	0.668	0.730	0.676	0.793	0.632
Mor	Tumor	0.656	0.656	0.808	0.553	0.526
	Stroma	0.746	0.843	0.761	0.946	0.857
	sTILs	0.766	0.817	0.763	0.879	0.750
	iTILs	0.562	0.719	0.639	0.821	0.625
	TILs	0.625	0.716	0.632	0.828	0.600
Clinical	ER	0.658	0.765	0.721	0.816	0.632
	ER%	0.668	0.776	0.702	0.868	0.667
	PR	0.669	0.795	0.700	0.921	0.750
	PR%	0.645	0.686	0.750	0.632	0.533
	HER2/CEP17 ratio	0.674	0.795	0.700	0.921	0.750
	Total Clinical	0.716	0.805	0.750	0.868	0.722
Tiles Count	Tumor	0.615	0.693	0.703	0.684	0.520
	Stroma	0.680	0.638	0.710	0.579	0.484
	sTILs	0.651	0.623	0.826	0.500	0.513
	iTILs	0.651	0.688	0.846	0.579	0.556
	TILs	0.596	0.760	0.627	0.721	0.666
Relative	TSR	0.576	0.769	0.660	0.921	0.667
	iTR	0.562	0.615	0.842	0.710	0.400
	sTR	0.571	0.758	0.632	0.947	0.600
	TISR	0.587	0.733	0.625	0.917	0.625
	LD	0.518	0.761	0.648	0.921	0.625
Latest Work	IMPRESS (H&E only) [27]	0.812	0.827	**0.906**	0.761	0.698
	Pathologists’ features [27]	0.788	0.782	0.870	0.711	0.645
	LTR [46]	0.544	0.758	0.632	0.947	0.600

Best-performed values are highlighted in boldface

Mor., morphological; TILs, tumor-infiltrating lymphocytes; iTILs, intratumoral TILs; sTILs, stromal TILs; PPV, positive predictive value; NPV, negative predictive value

The hybrid model based on sTILs-derived morphological and clinical features achieved the best performance, reaching an AUC of 0.873 and an F1 score of 0.889. Among clinical variables, the ER% yielded a high recall of 0.868 but moderate AUC (0.668), suggesting limited standalone discriminative power. Tile count comparisons showed that stromal tile count (AUC = 0.680) outperformed tumor tile count (AUC = 0.615), indicating stromal regions offer more predictive value. However, tile count models alone performed worse than hybrid models. Relative metrics such as TSR and TISR also showed limited discriminative ability, with AUCs ranging from 0.550 to 0.590. Detailed definitions and equations for these features are available in Additional File 2. Comprehensive external validation confirmed that features derived from stromal and sTIL regions, including both morphological and clinical data, achieved the strongest predictive performance for pCR. While some models performed well on individual metrics, none surpassed the hybrid model combining morphology and clinical features. Notably, the top-performing model exceeded pathologist-level accuracy, emphasizing the predictive value of stromal structure and immune infiltration in HER2 + BC treated with NAC.

**Fig. 4:**
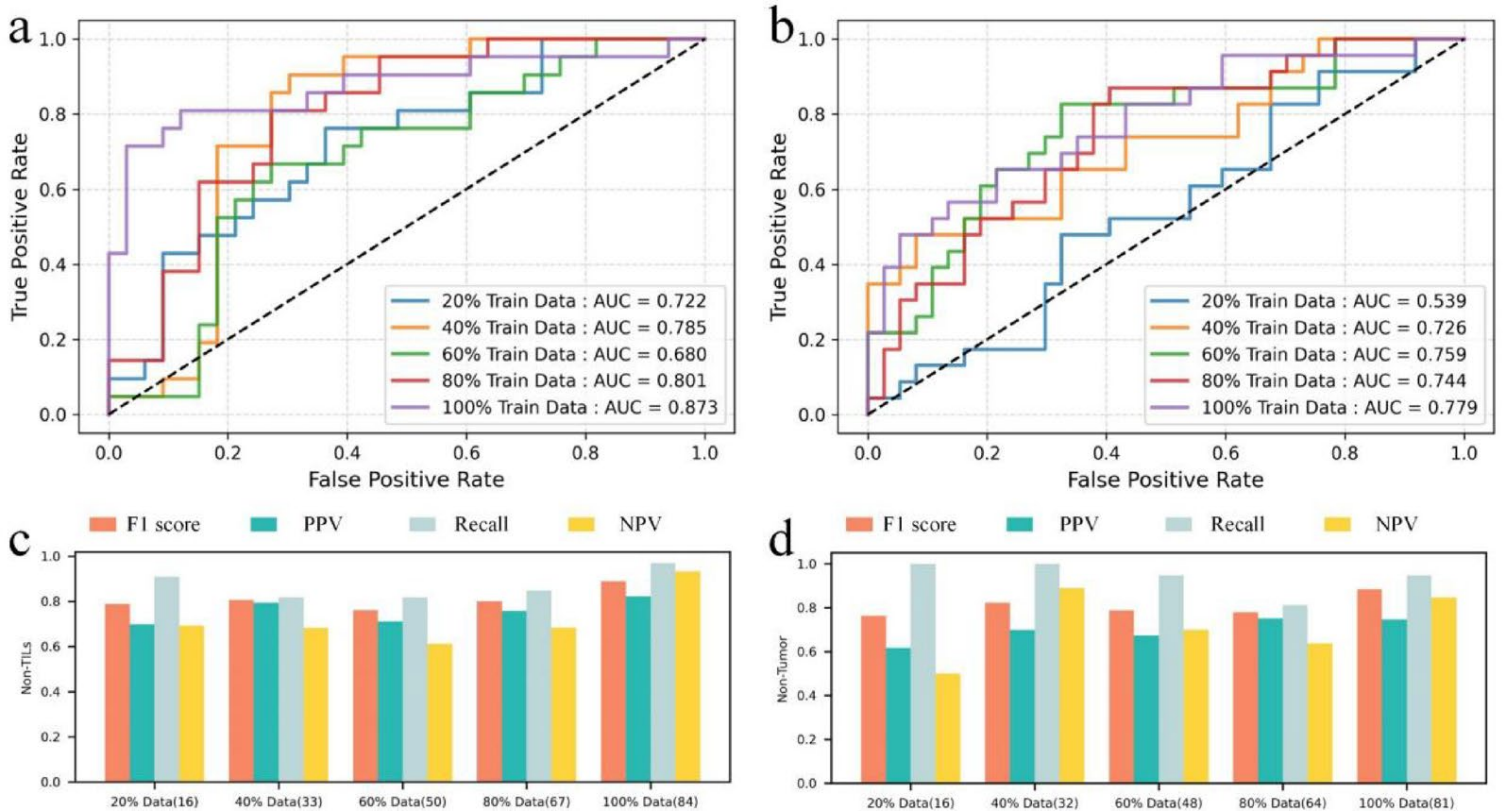
Validation of model generalization performance. **a** Receiver Operating Characteristic (ROC) curves for pCR prediction using both morphological and clinical features. **b** ROC curves for pCR prediction using only morphological features. **c** ROC curves based on varying training data proportions for sTILs, evaluated on a fixed external validation set. **d** ROC curves based on varying training data proportions for stroma, evaluated on a fixed external validation set. **e** Evaluation metrics (F1 score, PPV, recall, NPV) for sTILs at different training data proportions, all measured on the same external validation set. **f** Evaluation metrics (F1 score, PPV, recall, NPV) for stroma at different training data proportions, evaluated on the same external validation set

To validate the effectiveness of the proposed method, we compared its performance with recent studies. Table 3 shows that the model trained on morphological and clinical features of sTILs achieved the highest overall performance. In addition, the model based on stromal and sTIL-derived features outperformed the approach reported in reference [46]. Based on the evaluation summarized in Table 3 and Fig. 4a, b, the combined stroma and sTIL model demonstrated the strongest generalization ability, which informed our decision to focus subsequent analyses on these components.

Model generalizability was further assessed by varying the proportion of the training set while keeping the evaluation set fixed (IMPRESS HER2+ dataset). As illustrated in Fig. 4, performance improved with increasing training data and remained robust even with smaller subsets. Using only morphological features from sTILs regions in 20% of the training set, the model achieved an AUC of 0.722 on the external validation cohort, as shown in Fig. 4c. To assess model generalizability, we performed tenfold cross-validation and evaluated each fold’s model on the external validation set. The results showed consistent performance across folds in Additional File 2 (Table S6), indicating good robustness despite the limited training sample size. Throughout all subsets, sTIL-based models consistently outperformed stromal models, highlighting the predictive strength of immune infiltration patterns for pCR in patients receiving NAC.

### Feature importance analysis

**Fig. 5:**
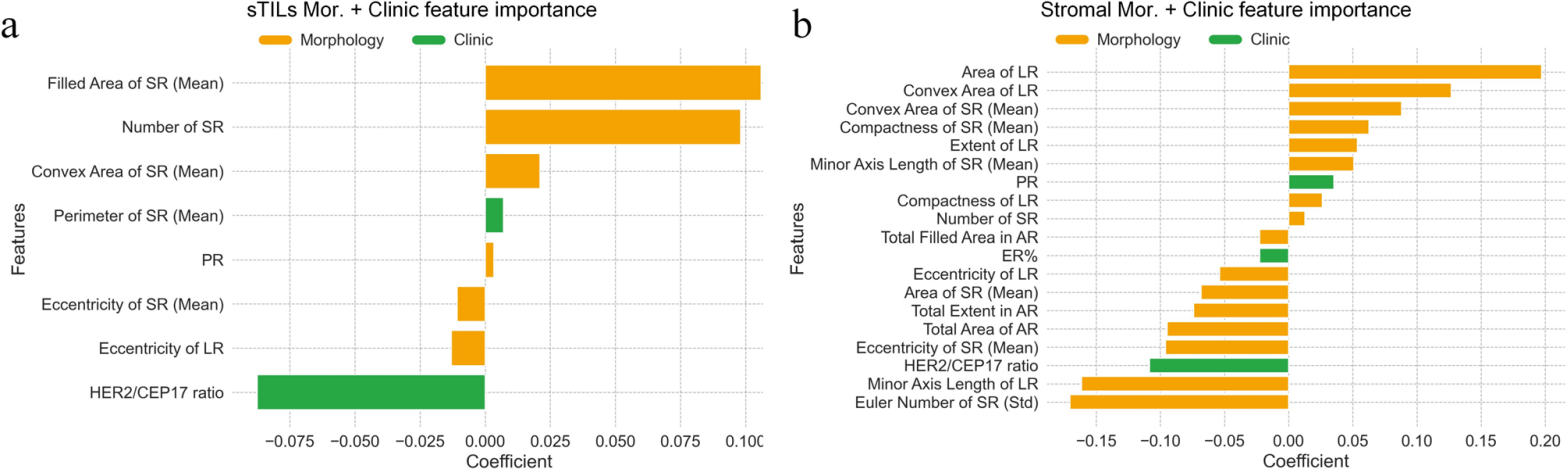
Feature importance selected by LASSO. **a** The importance of sTILs morphological and clinical combined features. **b** The importance of stromal morphological and clinical combined features. AR, all regions; SR, significant regions; LR, largest region

To better understand the contribution of region-specific morphological features from stroma and sTILs in combination with shared clinical variables, we analyzed the weights assigned to the LASSO-selected features. Figure 5 illustrates the distribution of these weights, and Additional File 2 (Table S7) lists the three most favorable and unfavorable features. In addition, we applied Spearman’s rank correlation test to assess correlations between paired features and detect potential dependencies. The results based on the Yale Response dataset and the IMPRESS HER2+ dataset are presented in Additional File 2 (Fig. S1 and S2).

Figure 5a illustrates spatial characteristics of the tumor immune microenvironment (TIME). The three most favorable features associated with significant regions are number of significant regions, filled area of significant regions (mean), and convex area of significant regions (mean). A higher number of significant regions in sTILs may indicate a stronger immune response to cancer cells and thus a better response to NAC. A greater filled area of significant regions (mean) suggests wider lymphocyte distribution, potentially contributing to a favorable pCR. Similarly, a larger convex area of significant regions (mean) reflects broader spatial spread or more complex margins, which may be linked to improved treatment outcomes. The most adverse features include HER2/CEP17 ratio, eccentricity of largest region, and eccentricity of significant regions (mean). Although HER2/CEP17 ratio is typically positively correlated with pCR, its negative weight in this model suggests that morphological features of sTILs may offer more predictive relevance. Eccentricity describes the shape of an ellipse and ranges from 0 to 1, where lower values indicate a more circular structure. Therefore, lymphocyte infiltration patterns with lower eccentricity may be more favorable for pCR prediction.

Figure 5b provides spatial information for the TME. The three most favorable features are the area of largest region, the convex area of largest region, and the convex area of significant regions (mean). A larger area may indicate a higher proportion of relevant immune cells. Higher values of the convex area of largest region and the convex area of significant regions (mean) suggest broader stromal cell distribution, which correlates positively with the likelihood of pCR. The three least predictive features are Euler number of significant regions (std), minor axis length of largest region, and HER2/CEP17 ratio. The Euler number measures spatial completeness by quantifying voids in the cavity region. A greater standard deviation of this metric across significant regions reflects variability in stromal connectivity, which may relate to differences in pCR outcomes. The minor axis length of largest region refers to the short axis of an ellipse, and smaller average values imply flatter morphological structures in stromal regions. This flatter configuration appears more favorable for pCR prediction.

In the stromal and sTILs regions, the TME and TIME show substantial spatial alignment, and the features identified in these regions display notable overlap, as illustrated in Fig. 5. PR, convex area of significant regions (mean), and number of significant regions are shared favorable features, while eccentricity of largest region, eccentricity of significant regions (mean), and HER2/CEP17 ratio are common unfavorable features. To further examine whether the most influential features selected by LASSO from the stroma and sTILs regions were independently associated with pCR after adjusting for potential confounding variables, we performed multivariate logistic regression analyses on the Yale Response dataset. Although not intended for predictive modeling, these analyses enhanced interpretability by quantifying the independent contribution of each feature. The detailed results, including odds ratios and 95% confidence intervals, are provided in Additional File 2 (Table S8 and Table S9).

### Univariate analysis

**Fig. 6:**
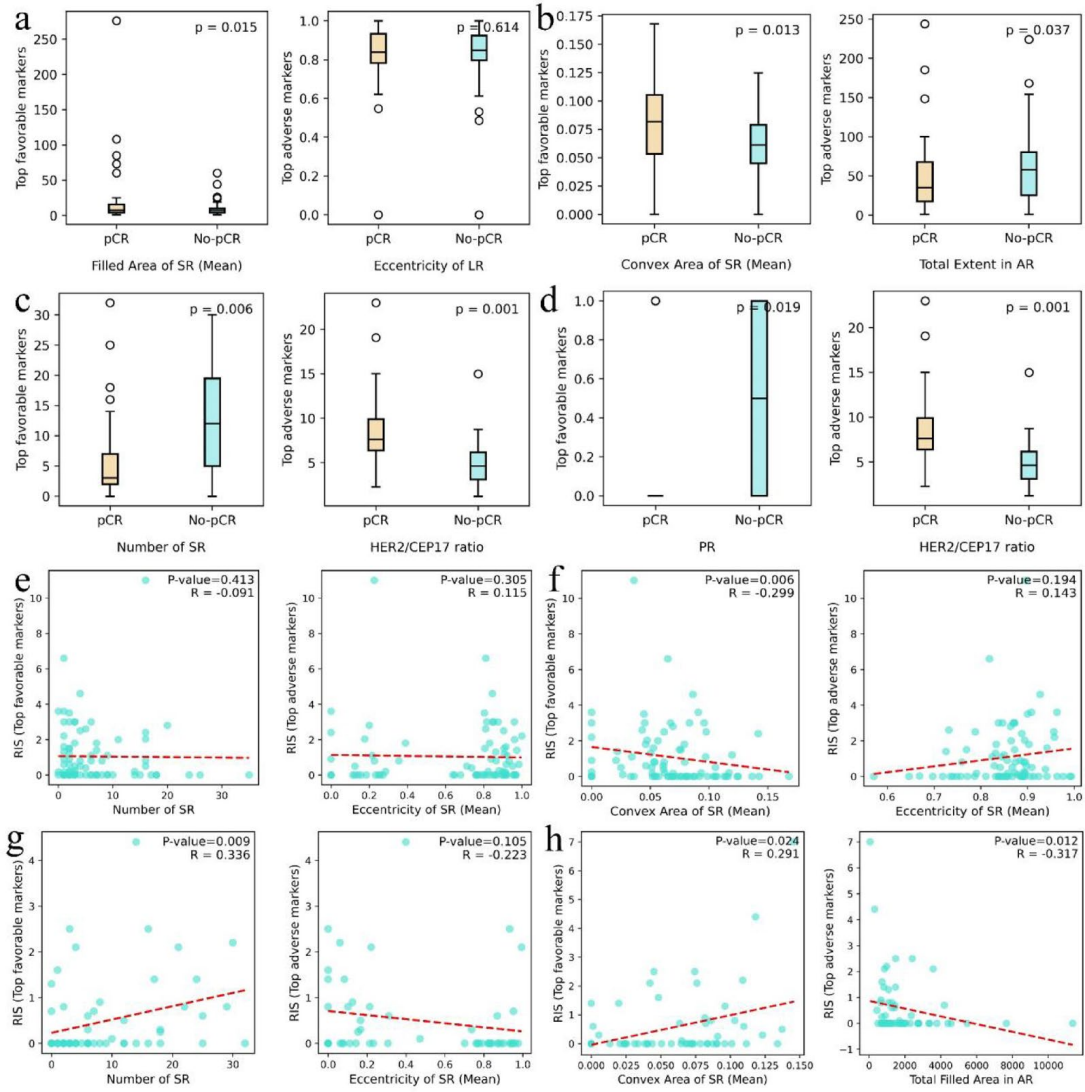
Univariate analysis results. **a**–**d** show the results of Mann–Whitney U tests assessing the association between individual features and pCR. a and b display results from sTILs and stroma in the Yale Response dataset, respectively, while c and d present findings from sTILs and stroma in the IMPRESS HER2+ dataset. **e**–**h** provide scatter plots showing spearman correlations (R) and corresponding *P* values between morphological features of sTILs and RIS. e and f correspond to sTILs and stroma in the Yale dataset, and **g** and **h** correspond to those in the IMPRESS HER2+ dataset. AR, all regions; SR, significant regions; LR, largest region

We performed statistical analysis to assess the relationship between stromal and sTILs features and pCR status. Group comparisons between pCR and non-pCR patients were conducted using Mann–Whitney U tests. From the features selected via LASSO, we identified the most influential favorable and adverse contributors in both tissue regions and further evaluated their distributions between clinical response groups. Figure 6 presents box plot comparisons, with full results available in Additional File 3.

In the Yale Response dataset (Fig. 6a, b), most features demonstrated significant intergroup differences except for eccentricity of largest region. These features include variables with both high favorable and adverse weights, as shown in Fig. 5. For example, the filled area of significant regions (mean) in the Yale dataset showed a *P* value of 0.015, and the number of significant regions in the IMPRESS HER2+ dataset had a *P* value of 0.006. Even features with relatively small weights, such as PR in the IMPRESS HER2 + cohort (*P* value = 0.008), showed discriminative potential. These findings suggest that individual features also contribute meaningfully to distinguishing between pCR and non-pCR.

In addition to the Mann–Whitney U test, we applied Spearman's rank correlation test to examine correlations between stroma and sTILs morphological features and RIS, where patients with pCR were assigned a RIS value of zero. In the Yale Response and IMPRESS HER2+ datasets, the median RIS values were 1.35 cm (range 0.02 to 11.00 cm) and 0.80 cm (range 0.10 to 7.00 cm), respectively. LASSO-selected features from both regions were further analyzed, and results are visualized through scatter plots in Fig. 6. Complete results are provided in Additional File 4. As shown in Fig. 6f, the mean convex area of significant regions in the stroma of the Yale dataset was negatively correlated with RIS (*P* value = 0.006, R = −0.299). In Fig. 6g, the number of significant regions in the sTILs of the IMPRESS HER2+ dataset showed a positive correlation (*P* value = 0.009, R = 0.336). These results suggest partially divergent correlation patterns across datasets, particularly for convex area of significant regions (mean), which demonstrated opposite correlation directions. This directional inconsistency likely reflects cohort-specific differences, including patient composition, TME, and imaging or segmentation conditions. In addition, features such as the number of significant regions (Fig. 6g) demonstrate potential for quantitative prediction of RIS.

## Discussion

Computational pathology methodologies have been widely employed in clinical applications to support diagnostic, outcome-related, and predictive objectives [47, 48]. Moreover, the analysis and evaluation of TME biomarkers in histopathological images of breast cancer can facilitate improved patient outcomes. To our knowledge, limited research has focused on predicting pCR to NAC in HER2 + BC patients through the quantification of TME morphological information. This study combines morphological features with clinical data to construct combined features, which are subsequently input into LASSO for feature selection. The selected features are then input into an MLP to predict the response to NAC treatment.

This research presents several notable advantages. Firstly, it addresses the challenge of analyzing large-scale digital histopathological images using ML algorithms. While researchers commonly process WSIs through tiling, variations in tile size can introduce information biases and lead to inconsistent model performance. This study employs DL to segment WSIs and generate TS-images, which serve as the basis for extracting spatial features of the TME, thereby replacing the original WSI. This strategy offers a more robust and reproducible approach to feature extraction compared with tile-level analysis, effectively reducing data complexity and minimizing noise. Secondly, the study conducts a comprehensive analysis by segmenting WSIs into five key regions, including tumor, stroma, iTILs, sTILs, and TILs. The findings demonstrate that combined morphological and clinical features derived from stroma and sTILs yield superior predictive performance for NAC response. Thirdly, although AI is often regarded as a promising clinical decision support tool, many DL-based studies suffer from limited interpretability and reproducibility. This research applies DL for foundational image segmentation on WSIs and subsequently employs image analysis techniques to extract interpretable features from all, significant and largest regions. In external validation, the top-performing model achieved an AUC of 0.873, surpassing various baseline models (Clinical, Tiles Count, and Relative), pathologists-level performance, and recently published state-of-the-art methods. Additionally, the study assesses the appropriateness of threshold selection for identifying significant regions and provides a comprehensive evaluation of interpretable morphological features from multiple perspectives, including the contributions of LASSO-selected features. Lastly, the paper addresses the common limitation of lacking external validation in existing studies due to the scarcity of publicly available NAC datasets. It incorporates the open-source Yale University dataset along with the IMPRESS HER2+ dataset for multicenter validation, confirming model robustness through percentage-based training. The experimental results demonstrate that the model exhibits strong generalizability when trained on combined morphological and clinical features from stroma and sTILs.

This study has several limitations. First, the relatively small sample size and the reliance on only two publicly available datasets, without an in-house validation cohort, may limit both the statistical robustness and the generalizability of our findings. The exclusive focus on a single molecular profile for predicting treatment response in HER2 + BC patients may also limit the generalizability of the results. Additionally, the proposed approach emphasizes morphological features due to their interpretability and computational efficiency, without incorporating other vision-based features such as Haralick texture descriptors or DL-based embeddings. While these omitted features may offer complementary information, they were not included in the present study. Future research could investigate hybrid feature representations that integrate DL-derived features with traditional descriptors to further improve predictive performance.

## Conclusions

Our study introduces a novel approach for predicting NAC response in HER2 + BC by integrating morphological and clinical features derived from histopathological images. Compared with traditional molecular biomarker methods, this approach is cost-effective, interpretable, and generalizable across independent datasets. DL techniques are first applied to segment the WSI into tumor, necrosis, and lymphocyte regions, generating TS-images. Morphological features are then extracted from tumor, stroma, iTILs, sTILs, and TILs, enabling a comprehensive characterization of TME and TIME. Integrating these features with clinical data significantly enhances pCR prediction performance, particularly with stroma and sTILs, outperforming recent comparable studies. This research provides an automated, accurate, and reproducible method for extracting spatial information from the TME with high clinical potential. Future studies will aim to validate the model on larger external cohorts and explore its application in other breast cancer subtypes and cancer types.

## Supplementary Information

Below is the link to the electronic supplementary material.

Additional file 1: Feature name.

Additional file 2: Supplementary methods and implementation details.

Additional file 3: Mann–Whitney U test results.

Additional file 4: Spearman-test_RIS results.

## Data Availability

No datasets were generated or analysed during the current study.

## Abbreviations

NAC: Neoadjuvant chemotherapy
HER2 + BC: HER2-positive breast cancer
HR+: Hormone receptor-positive
TNBC: Triple-negative breast cancer
pCR: Pathological complete response
TME: Tumor microenvironment
TIME: Tumor immune microenvironment
TS-images: Tissue segmentation images
TILs: Tumor-infiltrating lymphocytes
sTILs: Stromal tumor-infiltrating lymphocytes
iTILs: Intratumoral tumor-infiltrating lymphocytes
LASSO: Least absolute shrinkage and selection operator
AUC: Area under the curve
HER2+: Human epidermal growth factor receptor 2 positive
AI: Artificial intelligence
H&E: Hematoxylin and eosin
HER2CN: HER2 copy number
IHC: Immunohistochemistry
ER+: Estrogen receptor-positive
PR+: Progesterone receptor-positive
ML: Machine learning
WSIs: Whole slide images
T-CNN: CNN for tumor classification
N-CNN: CNN for necrosis classification
L-CNN: CNN for lymphocytes infiltrating classification
GPL: Global pooling layer
HER2/CEP17 ratio: The ratio of HER2 gene copy number to chromosome 17 centromere signals
PPV: Positive predictive value
NPV: Negative predictive value
RIS: Residual infiltration size
Mor.: Morphological features
ROC: Receiver operating characteristic
AR: All Regions
SR: Significant regions
LR: Largest region
