# Supplementary material for: Morphological analysis of tumor microenvironment in HER2-positive breast cancer: predicting response to neoadjuvant chemotherapy on histopathological images
Source: Breast Cancer Res. 2025 Oct 21;27:182. doi: 10.1186/s13058-025-02139-x (PMC12542099; doi:10.1186/s13058-025-02139-x)
Supplement: Supplementary file 2 — Additional file 2: Supplementary methods and implementation details. [file 13058_2025_2139_MOESM2_ESM.docx]

Deep learning for tissue region identification

Tumor classification

We analyzed 195 whole slide images (WSIs) at 40× magnification from The Cancer Genome Atlas, using corresponding annotation masks provided by Angel Cruz-Roa et al. [1]. These annotation masks, derived from expert pathologist labels, delineated tumor regions at the pixel level and were aligned with the WSIs to assign each tile a label of either tumor or stroma. WSIs were segmented into tile images with dimensions of 350×350×3 pixels. Otsu’s thresholding was applied to exclude tiles with less than 26 percent tissue content. Tumor regions were annotated based on the mask images that delineated tumor areas. Stroma was defined as non-fat tissue located outside these annotated tumor regions within core needle biopsy pathology images, excluding non-informative areas eliminated during preprocessing. Spatial coordinates from the annotation masks were aligned with the WSIs to assign each tile a label of either tumor or stroma. This process resulted in 2,260,684 labeled tiles. The 195 patients were split into training, validation, and test sets. The training set consisted of 157 patients, accounting for 1,708,303 tiles; the validation set included 18 patients with 201,135 tiles; and the test set contained 20 patients with 303,019 tiles. To alleviate computational demands, we randomly selected 20% of the tiles from each patient in the training set, yielding a total of 351,246 tiles. Tumor versus stroma classification was conducted using a modified Xception model adapted via transfer learning [2]. The final fully connected layer was replaced by a global max pooling layer and two output neurons with SoftMax activation, enhancing input flexibility, reducing model parameters, and improving generalization. The model was trained using stochastic gradient descent with a learning rate of 0.01, a momentum of 0.9, a batch size of 128, and the cross-entropy loss function.

Necrosis classification

To reduce false positives arising from morphological similarities between necrotic cell nuclei and intratumoral tumor-infiltrating lymphocytes, we developed a convolutional neural network (CNN) for necrosis identification. The dataset comprised 2,237 image tiles (500×500×3 pixels) from LUAD cases at 20× magnification, sourced from a publicly available database [3]. To enhance robustness and generalizability, we employed a sliding window approach with 100×100 pixel windows and a stride of 100 pixels. This process yielded a total of 55,925 tiles. A tile was labeled as necrotic if more than 20 percent of its pixels contained necrotic tissue [4]. A fine-tuned VGG-16 architecture was employed for transfer learning[5]. The network was optimized by substituting the fully connected layers with a global max pooling layer, a dropout layer with a rate of 0.5, a dense layer of 128 neurons activated by ReLU, and a SoftMax output layer. Training was performed using the Adam optimizer with a learning rate of 0.0001, a batch size of 16, and 100 training epochs. Model evaluation was performed using stratified five-fold cross-validation, with each fold consisting of 44,740 training tiles and 11,185 testing tiles

Lymphocytes classification

To analyze the tumor immune microenvironment, we implemented a deep learning model for lymphocyte identification. This model was trained and validated using public datasets of LUAD (10 patients) and BRCA (18 patients) at 20× magnification, totaling 34,404 tiles, each with a resolution of 500×500×3 pixels [5]. Lymphocyte infiltration status was determined based on the central 100×100 pixel region of each tile. Tiles were labeled as infiltrated if lymphocytes were visibly present within the central 100×100 pixel region, based on expert annotation. Stratified five-fold cross-validation was conducted, with each fold consisting of 27,523 training tiles and 6,881 testing tiles. The model employed the same architecture and hyperparameters as used for necrosis detection (fine-tuned VGG-16, Adam optimizer, learning rate = 0.0001, batch size = 16, 100 epochs)

Tissue region identification results

During the generation of tissue segment images, we developed and trained three separate models, each dedicated to a specific classification task. The modeling procedure for each task is described as follows.

For tumor classification, the Xception architecture was subjected to full-layer fine-tuning with an early stopping strategy to prevent overfitting. Training converged at epoch 25. Evaluation on the validation and test sets yielded classification accuracies of 0.865 and 0.860, respectively, with corresponding area under the curve (AUC) values of 0.927 and 0.917. The stable performance across datasets demonstrates strong generalizability and validates the sampling approach. To address magnification inconsistencies between the Yale Response and IMPRESS HER2-positive datasets (20×) and the model’s requirement for 40× input resolution, neoadjuvant chemotherapy (NAC) images were upsampled from 175×175×3 to 350×350×3 using bidirectional interpolation.

The VGG-16 model was employed for necrosis classification and was similarly fine-tuned. Stratified five-fold cross-validation produced a mean classification accuracy of 0.964±0.035. Final predictions were generated through ensemble averaging of probabilities across the five validation folds. The same modeling framework was applied for lymphocyte classification, resulting in a mean accuracy of 0.914 ± 0.031. Final outputs were again derived by averaging the predictions from the cross-validated models.

Relative feature

**Tumor-to-Stroma Ratio (TSR)**

$$\begin{aligned} TSR=\frac{Tumor Tile Counts}{Stroma Tile Counts+Tumor Tile Counts}\#\left( 1 \right) \end{aligned}$$

**iTILs-to-Tumor Ratio (iTR)**

$$\begin{aligned} iTR=\frac{iTILs Tile Counts}{Tumor Tile Counts+iTILs Tile Counts}\#\left( 2 \right) \end{aligned}$$

**sTILs Ratio(sTR)**

$$\begin{aligned} sTR=\frac{sTILs Tile Counts}{Stroma Tile Counts+sTILs Tile Counts}\#\left( 3 \right) \end{aligned}$$

**TILs-to-Stroma Ratio (TISR)**

$$\begin{aligned} TISR=\frac{TILs Tile Counts}{TILs Tile Counts+Stroma Counts}\#\left( 4 \right) \end{aligned}$$

**Lymphocyte Density (LD)**

$$\begin{aligned} LD=\frac{TILs Tile Counts}{ToTal Tissue Counts}\#\left( 5 \right) \end{aligned}$$

Table S1. Ten-fold cross-validation performance on the training set across different significance thresholds in each region using morphological and clinical features.

| Region | Sign. thresholds | AUC | F1 score | PPV | Recall | NPV |
| --- | --- | --- | --- | --- | --- | --- |
| Tumor | 1% | 0.618±0.055 | 0.626±0.054 | 0.514±0.059 | 0.825±0.130 | 0.692±0.070 |
|  | 3% | **0.633±0.073** | **0.648±0.059** | 0.508±0.076 | **0.921±0.103** | 0.669±0.216 |
|  | 5% | 0.623±0.097 | 0.637±0.068 | 0.512±0.069 | 0.875±0.147 | **0.713±0.076** |
|  | 10% | 0.602±0.057 | 0.644±0.044 | **0.517±0.057** | 0.869±0.094 | 0.681±0.125 |
| Stroma | 1% | 0.630±0.062 | 0.615±0.067 | 0.534±0.065 | 0.740±0.125 | 0.674±0.087 |
|  | 3% | 0.665±0.053 | 0.603±0.051 | **0.568±0.082** | 0.669±0.125 | 0.674±0.050 |
|  | 5% | **0.686±0.068** | **0.648±0.067** | **0.568±0.082** | 0.795±0.164 | **0.733±0.068** |
|  | 10% | 0.668±0.080 | 0.641±0.068 | 0.538±0.087 | **0.833±0.159** | 0.703±0.112 |
| iTILs | 1% | **0.639±0.078** | **0.641±0.052** | 0.514±0.075 | **0.885±0.119** | 0.701±0.078 |
|  | 3% | 0.631±0.093 | 0.626±0.061 | 0.514±0.081 | 0.846±0.156 | 0.655±0.170 |
|  | 5% | 0.624±0.061 | 0.636±0.079 | 0.518±0.075 | 0.857±0.181 | **0.718±0.106** |
|  | 10% | 0.622±0.115 | 0.631±0.081 | **0.525±0.100** | 0.834±0.159 | 0.590±0.184 |
| sTILs | 1% | 0.608±0.086 | 0.605±0.061 | 0.502±0.078 | 0.821±0.186 | 0.623±0.202 |
|  | 3% | 0.637±0.090 | 0.612±0.069 | 0.503±0.076 | **0.843±0.204** | 0.655±0.121 |
|  | 5% | **0.701±0.111** | **0.674±0.077** | **0.615±0.127** | 0.786±0.115 | **0.693±0.084** |
|  | 10% | 0.675±0.058 | 0.626±0.037 | 0.539±0.074 | 0.810±0.185 | 0.657±0.084 |
| TILs | 1% | 0.639±0.075 | 0.629±0.055 | 0.536±0.111 | **0.828±0.165** | 0.644±0.158 |
|  | 3% | 0.629±0.093 | 0.641±0.059 | 0.568±0.137 | 0.818±0.168 | 0.586±0.257 |
|  | 5% | **0.651±0.060** | 0.620±0.007 | 0.543±0.076 | 0.767±0.143 | **0.657±0.084** |
|  | 10% | 0.640±0.064 | **0.658±0.039** | **0.602±0.087** | 0.777±0.174 | 0.586±0.056 |

Best-performed values are highlighted in boldface. Sign.: significance, TILs: tumor infiltrating lymphocytes, iTILs: intratumoral TILs, sTILs: stromal TILs.

Table S2. Ten-fold cross-validation performance on the training set across different significance thresholds in each region using morphological features.

| Region | Sign. thresholds | AUC | F1 score | PPV | Recall | NPV |
| --- | --- | --- | --- | --- | --- | --- |
| Tumor | 1% | 0.633±0.053 | 0.644±0.041 | **0.523±0.062** | 0.861±0.115 | **0.719±0.052** |
|  | 3% | **0.671±0.054** | 0.640±0.038 | 0.517±0.057 | 0.861±0.091 | 0.700±0.031 |
|  | 5% | 0.635±0.098 | 0.630±0.078 | 0.507±0.074 | 0.870±0.172 | 0.709±0.096 |
|  | 10% | 0.608±0.082 | **0.646±0.057** | 0.521±0.067 | **0.874±0.131** | 0.699±0.096 |
| Stroma | 1% | 0.611±0.060 | 0.619±0.049 | 0.503±0.065 | **0.835±0.124** | 0.667±0.096 |
|  | 3% | 0.640±0.079 | 0.632±0.054 | 0.541±0.082 | 0.791±0.124 | 0.691±0.065 |
|  | 5% | **0.663±0.075** | **0.647±0.054** | **0.585±0.123** | 0.771±0.136 | **0.714±0.052** |
|  | 10% | 0.643±0.085 | 0.576±0.083 | 0.539±0.123 | 0.643±0.074 | 0.638±0.074 |
| iTILs | 1% | 0.628±0.064 | **0.660±0.038** | 0.535±0.063 | **0.890±0.123** | **0.744±0.049** |
|  | 3% | **0.656±0.077** | 0.636±0.050 | 0.518±0.087 | 0.861±0.111 | 0.661±0.177 |
|  | 5% | 0.625±0.087 | 0.643±0.069 | **0.545±0.088** | 0.829±0.167 | 0.718±0.095 |
|  | 10% | 0.628±0.096 | 0.636±0.062 | 0.520±0.080 | 0.857±0.145 | 0.608±0.163 |
| sTILs | 1% | 0.621±0.091 | 0.656±0.059 | 0.511±0.067 | **0.937±0.105** | **0.709±0.161** |
|  | 3% | 0.604±0.070 | 0.620±0.032 | 0.474±0.050 | 0.921±0.107 | 0.625±0.185 |
|  | 5% | **0.683±0.129** | **0.668±0.096** | **0.602±0.127** | 0.807±0.176 | 0.650±0.238 |
|  | 10% | 0.620±0.064 | 0.626±0.073 | 0.484±0.043 | 0.910±0.161 | 0.674±0.078 |
| TILs | 1% | **0.672±0.073** | 0.676±0.048 | 0.538±0.058 | **0.929±0.104** | **0.753±0.078** |
|  | 3% | 0.659±0.099 | 0.637±0.057 | **0.577±0.136** | 0.792±0.173 | 0.632±0.191 |
|  | 5% | 0.627±0.074 | 0.621±0.047 | 0.512±0.090 | 0.838±0.142 | 0.629±0.177 |
|  | 10% | 0.610±0.055 | **0.677±0.064** | 0.565±0.069 | 0.857±0.105 | 0.555±0.178 |

Best-performed values are highlighted in boldface. Sign.: significance, TILs: tumor infiltrating lymphocytes, iTILs: intratumoral TILs, sTILs: stromal TILs.

Table S3. Generalization performance of morphological features across five tissue types under varying significant region thresholds in the IMPRESS_HER2+ validation set.

| Region | Sign. thresholds | AUC | F1 score | PPV | Recall | NPV |
| --- | --- | --- | --- | --- | --- | --- |
| Tumor | 1% | 0.637 | **0.783** | 0.667 | **0.947** | **0.700** |
|  | 3% | 0.657 | 0.756 | 0.705 | 0.816 | 0.600 |
|  | 5% | 0.656 | 0.656 | **0.808** | 0.553 | 0.526 |
|  | 10% | **0.658** | 0.756 | 0.654 | 0.895 | 0.583 |
| Stroma | 1% | 0.639 | 0.804 | 0.685 | **0.974** | 0.800 |
|  | 3% | 0.689 | 0.783 | 0.667 | 0.947 | 0.700 |
|  | 5% | **0.746** | **0.843** | **0.761** | 0.946 | **0.857** |
|  | 10% | 0.667 | 0.746 | 0.688 | 0.815 | 0.571 |
| iTILs | 1% | 0.521 | **0.766** | **0.643** | **0.947** | **0.625** |
|  | 3% | 0.548 | 0.719 | 0.582 | 0.941 | 0.400 |
|  | 5% | 0.562 | 0.719 | 0.639 | 0.821 | **0.625** |
|  | 10% | **0.567** | 0.632 | 0.600 | 0.667 | 0.553 |
| sTILs | 1% | 0.689 | 0.767 | 0.673 | **0.892** | 0.615 |
|  | 3% | 0.671 | 0.750 | 0.652 | 0.882 | 0.615 |
|  | 5% | **0.766** | **0.817** | **0.763** | 0.879 | **0.750** |
|  | 10% | 0.565 | 0.467 | 0.583 | 0.389 | 0.571 |
| TILs | 1% | 0.582 | **0.750** | 0.660 | 0.868 | 0.571 |
|  | 3% | 0.607 | 0.729 | 0.608 | **0.912** | 0.556 |
|  | 5% | **0.625** | 0.716 | **0.632** | 0.828 | 0.600 |
|  | 10% | 0.616 | 0.703 | 0.591 | 0.867 | **0.700** |

Best-performed values are highlighted in boldface. Sign.: significance, TILs: tumor infiltrating lymphocytes, iTILs: intratumoral TILs, sTILs: stromal TILs.

Table S4. Performance comparison of different classifiers using LASSO-selected morphological and clinical features across regions.

| Region | Classifier | AUC | F1 score | PPV | Recall | NPV |
| --- | --- | --- | --- | --- | --- | --- |
| Tumor | MLP | **0.732** | **0.740** | **0.771** | 0.711 | 0.586 |
|  | SVM | 0.611 | 0.701 | 0.692 | 0.711 | 0.520 |
|  | LR | 0.729 | 0.762 | 0.696 | **0.842** | **0.611** |
|  | RF | 0.578 | 0.675 | 0.643 | 0.711 | 0.455 |
| Stroma | MLP | **0.779** | **0.883** | **0.745** | 0.946 | 0.846 |
|  | SVM | 0.607 | 0.711 | 0.692 | 0.730 | 0.522 |
|  | LR | 0.759 | 0.831 | 0.712 | **1.000** | **0.900** |
|  | RF | 0.667 | 0.787 | 0.649 | 0.812 | 0.800 |
| sTILs | MLP | **0.873** | **0.889** | **0.821** | **0.970** | **0.933** |
|  | SVM | 0.859 | 0.795 | 0.689 | 0.939 | 0.727 |
|  | LR | 0.869 | 0.833 | 0.769 | 0.909 | 0.765 |
|  | RF | 0.743 | 0.789 | 0.737 | 0.848 | 0.667 |
| iTILs | MLP | **0.594** | 0.654 | **0.708** | 0.607 | 0.571 |
|  | SVM | 0.552 | 0.718 | 0.560 | 0.723 | 0.500 |
|  | LR | 0.576 | 0.511 | 0.632 | 0.429 | 0.485 |
|  | RF | 0.510 | **0.720** | 0.574 | **0.764** | **0.600** |
| TILs | MLP | **0.668** | 0.730 | **0.676** | 0.793 | 0.632 |
|  | SVM | 0.635 | 0.722 | 0.605 | **0.897** | 0.600 |
|  | LR | 0.635 | 0.719 | 0.657 | 0.793 | 0.611 |
|  | RF | 0.522 | **0.743** | 0.634 | 0.797 | **0.667** |

Best-performed values are highlighted in boldface. Sign.: significance, TILs: tumor infiltrating lymphocytes, iTILs: intratumoral TILs, sTILs: stromal TILs.

Table S5. Performance comparison of different classifiers using LASSO-selected morphological features across regions.

| Region | Classifier | AUC | F1 score | PPV | Recall | NPV |
| --- | --- | --- | --- | --- | --- | --- |
| Tumor | MLP | 0.656 | 0.656 | **0.808** | 0.553 | **0.526** |
|  | SVM | 0.659 | **0.701** | 0.692 | **0.711** | 0.520 |
|  | LR | **0.667** | 0.648 | 0.697 | 0.605 | 0.484 |
|  | RF | 0.529 | 0.609 | 0.677 | 0.553 | 0.455 |
| Stroma | MLP | **0.746** | **0.843** | **0.761** | 0.946 | 0.857 |
|  | SVM | 0.706 | 0.786 | 0.702 | 0.892 | 0.667 |
|  | LR | 0.672 | 0.791 | 0.694 | 0.919 | 0.692 |
|  | RF | 0.636 | 0.822 | 0.698 | **1.000** | **0.889** |
| sTILs | MLP | 0.766 | **0.817** | **0.763** | 0.879 | **0.750** |
|  | SVM | 0.690 | 0.789 | 0.698 | 0.909 | 0.692 |
|  | LR | **0.768** | 0.811 | 0.732 | 0.909 | 0.733 |
|  | RF | 0.733 | 0.775 | 0.660 | **0.939** | 0.667 |
| iTILs | MLP | **0.562** | 0.719 | **0.639** | **0.821** | **0.625** |
|  | SVM | 0.516 | 0.516 | 0.600 | 0.514 | 0.452 |
|  | LR | 0.503 | 0.490 | 0.571 | 0.429 | 0.452 |
|  | RF | 0.528 | **0.720** | 0.574 | 0.764 | 0.600 |
| TILs | MLP | **0.625** | 0.716 | **0.632** | 0.828 | **0.600** |
|  | SVM | 0.553 | 0.722 | 0.605 | 0.897 | **0.600** |
|  | LR | 0.562 | 0.712 | 0.591 | 0.792 | 0.556 |
|  | RF | 0.609 | **0.718** | 0.571 | **0.966** | 0.500 |

Best-performed values are highlighted in boldface. Sign.: significance, TILs: tumor infiltrating lymphocytes, iTILs: intratumoral TILs, sTILs: stromal TILs.

Table S6. Performance metrics (mean ± std) of the model evaluated on training and external validation sets using 10-fold cross-validationt

| Region | Dataset Type | | AUC | | F1 score | | PPV | | Recall | | NPV |
| --- | --- | --- | --- | --- | --- | --- | --- | --- | --- | --- | --- |
| sTILs | Train Set | | 0.701±0.111 | | 0.674±0.077 | | 0.615±0.127 | | 0.786±0.115 | | 0.693±0.084 |
|  | ValidationSet | | 0.756±0.082 | | 0.787±0.015 | | 0.701±0.053 | | 0.809±0.069 | | 0.692±0.074 |
| Stroma | Train Set | 0.686±0.068 | | 0.648±0.067 | | 0.568±0.082 | | 0.795±0.164 | | 0.733±0.068 | |
|  | ValidationSet | 0.737±0.046 | | 0.823±0.018 | | 0.710±0.017 | | 0.878±0.020 | | 0.836±0.068 | |

Train Set: Yale Response Dataset, ValidationSet : IMPRESS HER2+ Dataset.

Table S7. Feature weights for stromal and stromal tumor-infiltrating lymphocyte (sTIL) features in the Yale Response Dataset. (SR: Significant Regions; LR: Largest Region; Coef. coefficient.)

| Cohort | Favorable Features | | | Adverse Features | | |
| --- | --- | --- | --- | --- | --- | --- |
|  | Rank | Feature Name | Coef. | Rank | Feature Name | Coef. |
| sTILs | 1 | Number of SR | 0.101 | 1 | HER2/CEP17 ratio | -0.089 |
|  | 2 | Filled Area of SR (Mean) | 0.088 | 2 | Eccentricity of LR | -0.016 |
|  | 3 | Convex Area of SR (Mean) | 0.028 | 3 | Eccentricity of SR (Mean) | -0.011 |
| Stroma | 1 | Area of LR | 0.193 | 1 | Euler Number of SR (Std) | -0.170 |
|  | 2 | Convex Area of LR | 0.131 | 2 | Minor Axis Length of LR | -0.158 |
|  | 3 | Convex Area of SR (Mean) | 0.092 | 3 | HER2/CEP17 ratio | -0.101 |

Table S8.Multivariate logistic regression analysis of LASSO-selected morphological and clinical features from the sTILs region in the Yale Response Dataset.

| Variable | Coefficient | OR | CI 2.5% | CI 97.5% | P value |
| --- | --- | --- | --- | --- | --- |
| HER2/CEP17 ratio | -0.685 | 0.504 | 0.274 | 0.928 | **0.028** |
| Number of SR | 0.660 | 1.934 | 0.911 | 4.105 | **0.036** |
| Filled Area of SR (Mean) | 0.672 | 1.958 | 0.637 | 6.016 | 0.241 |
| Convex Area of SR (Mean) | 0.316 | 1.371 | 0.725 | 2.594 | 0.332 |
| Perimeter of SR (Mean) | -0.257 | 0.773 | 0.458 | 1.304 | 0.357 |
| Eccentricity of LR | 0.450 | 1.569 | 0.601 | 4.095 | 0.410 |
| PR | -0.270 | 0.763 | 0.402 | 1.451 | 0.480 |
| Eccentricity of SR (Mean) | 0.186 | 1.205 | 0.719 | 2.020 | 0.765 |

SR: Significant Regions, LR: Largest Region. Bolded P values indicate statistical significance (P value < 0.05).

Table S9.Multivariate logistic regression analysis of LASSO-selected morphological and clinical features from the stroma region in the Yale Response Dataset.

| Variable | Coefficient | OR | CI 2.5% | CI 97.5% | P value |
| --- | --- | --- | --- | --- | --- |
| Minor Axis Length of LR | -4.754 | 0.009 | 0.000 | 0.234 | **0.002** |
| Area of LR | 2.640 | 14.00 | 4.550 | 43.16 | **0.004** |
| Euler Number of SR (Std) | -3.975 | 0.019 | 0.001 | 0.283 | **0.005** |
| HER2/CEP17 ratio | -1.125 | 0.325 | 0.116 | 0.906 | **0.013** |
| Total Filled Area in AR | -0.213 | 0.808 | 0.673 | 0.970 | **0.021** |
| Total Area of AR | 1.204 | 3.334 | 1.092 | 10.214 | **0.029** |
| Eccentricity of LR | -1.239 | 0.29 | 0.076 | 1.101 | **0.040** |
| Minor Axis Length of SR (Mean) | -5.46 | 0.004 | 0.000 | 0.128 | **0.045** |
| Area of SR (Mean) | -0.900 | 0.407 | 0.138 | 1.199 | 0.055 |
| Convex Area of SR (Mean) | 0.728 | 2.071 | 0.821 | 5.226 | 0.080 |
| PR | 0.534 | 1.706 | 0.736 | 3.956 | 0.106 |
| Eccentricity of SR (Mean) | -0.574 | 0.563 | 0.206 | 1.536 | 0.123 |
| Number of SR | 0.472 | 1.603 | 0.467 | 5.503 | 0.127 |
| Compactness of LR | 0.571 | 1.77 | 0.223 | 14.039 | 0.163 |
| Compactness of SR (Mean) | 0.544 | 1.723 | 0.639 | 4.640 | 0.176 |
| Extent of LR | 0.496 | 1.643 | 0.518 | 5.207 | 0.674 |
| Total Extent in AR | -0.788 | 0.455 | 0.079 | 2.605 | 0.733 |
| ER% | -0.198 | 0.821 | 0.393 | 1.712 | 0.786 |
| Convex Area of LR | 0.871 | 2.390 | 1.051 | 5.432 | 0.796 |

AR: All Regions, SR: Significant Regions, LR: Largest Region. Bolded P values indicate statistical significance (P value < 0.05).

To address potential confounding and evaluate the independence of selected features, we conducted multivariate logistic regression analyses using the Yale Response dataset. The analysis was designed to assess whether morphological and clinical variables selected by LASSO retained statistically significant associations with pCR after adjusting for potential collinearity. Separate models were built for features derived from the sTILs and stroma regions.

As shown in Supplementary Tables S7 and S8, several features demonstrated independent associations with pCR. In the model based on sTILs-derived features, the HER2/CEP17 ratio (P value = 0.028) and the number of significant regions (P value = 0.036) were statistically significant. Other variables such as filled area of SR and eccentricity of SR showed positive coefficients but did not reach statistical significance. In the model based on stroma-derived features, multiple morphological and clinical variables remained significant. These included the minor axis length of the largest region (P value = 0.002), area of the largest region (P value = 0.004), and the standard deviation of the Euler number of significant regions (P value = 0.005). Additionally, the HER2/CEP17 ratio (P value = 0.013) and the total filled area in the annotated region (P value = 0.021) also reached statistical significance, suggesting their independent association with pCR in the presence of other covariates.

To further evaluate generalizability, we tested these features on the independent IMPRESS-HER2+ dataset. External validation showed that the model based on sTILs features achieved an AUC of 0.864 and a peak F1 score of 0.769. The model based on stroma features yielded an AUC of 0.839 and a peak F1 score of 0.694. These results support the robustness and reproducibility of the selected features and their relevance across datasets.

**Correlation analysis between features**


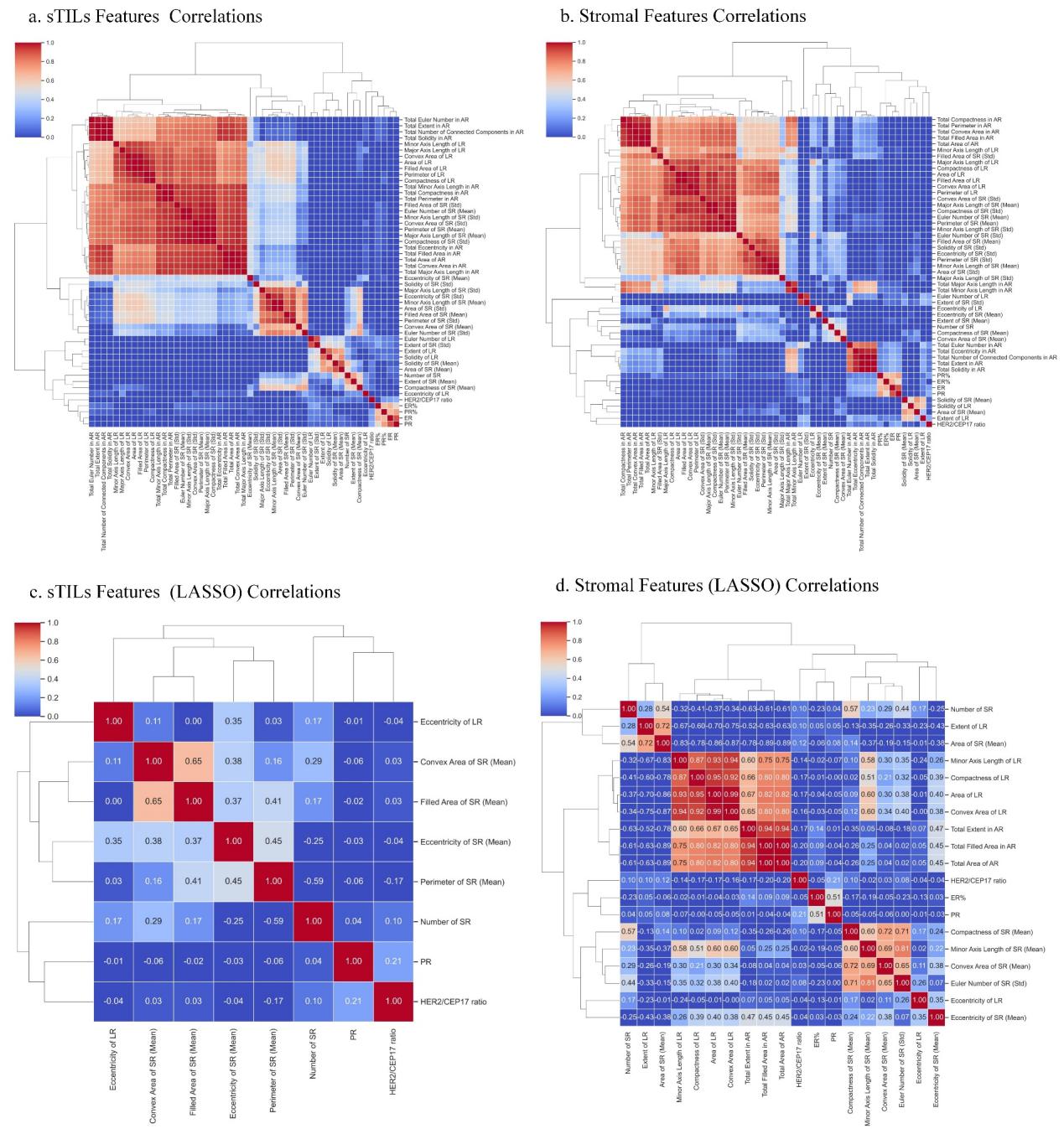


Fig. S1. Spearman correlation analysis of stromal and stromal tumor-infiltrating lymphocytes (sTIL) features in the Yale Response Dataset. a. Correlation heatmap of morphological and clinical features of sTILs. b. Correlation heatmap of morphological and clinical features of stroma. c. Correlation heatmap of LASSO-selected morphological and clinical features of sTILs. d. Correlation heatmap of LASSO-selected morphological and clinical features of stroma. Mor.: Morphological features, AR: All Region, SR: Significant Regions, LR: Largest Region.. Viewing in color is recommended.


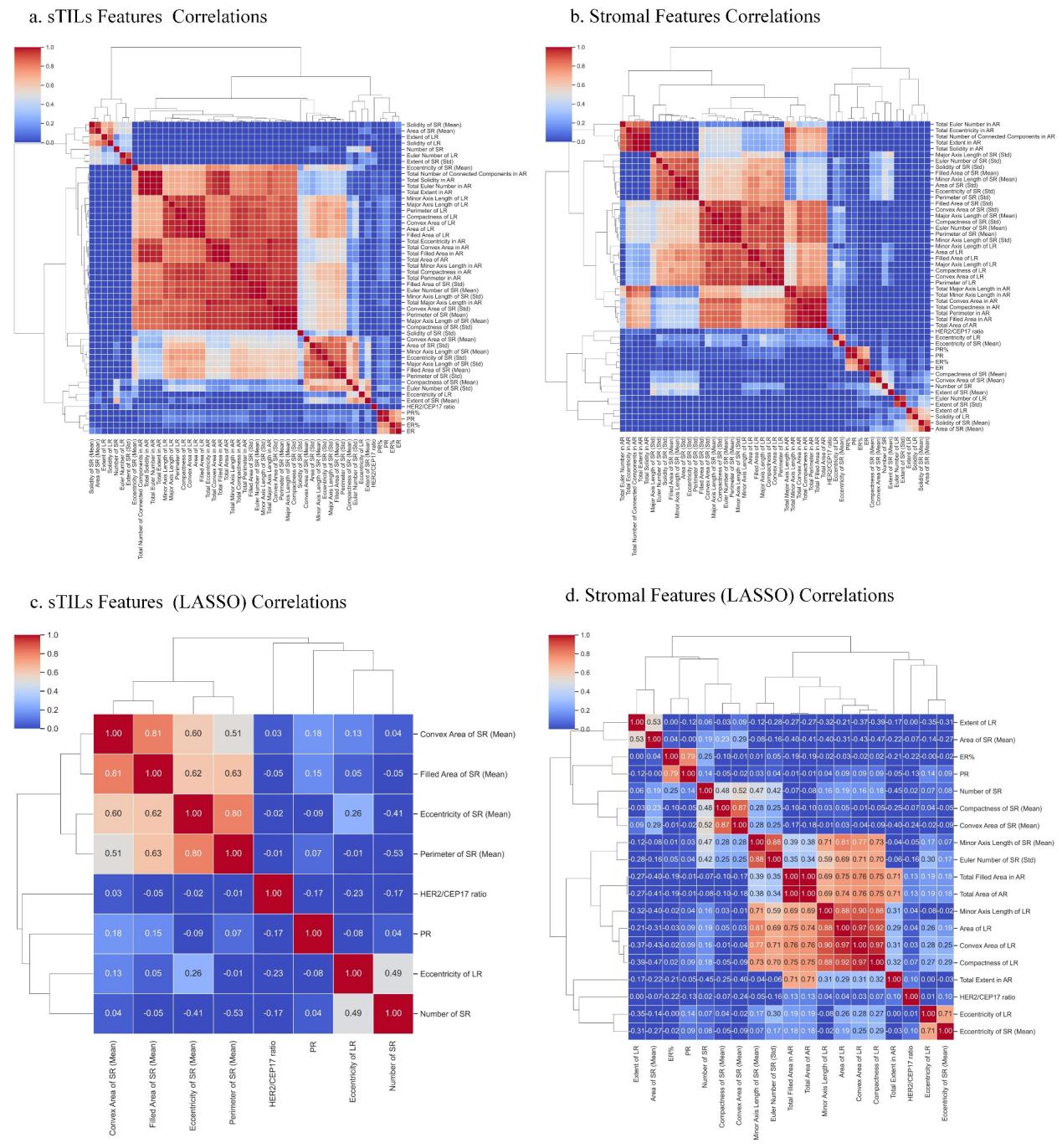


Fig. S2. Spearman correlation analysis of stromal and stromal tumor-infiltrating lymphocytes (sTIL) features in IMPRESS HER2+ Dataset. a. Correlation heatmap of morphological and clinical features of sTILs. b. Correlation heatmap of morphological and clinical features of stroma. c. Correlation heatmap of LASSO-selected morphological and clinical features of sTILs. d. Correlation heatmap of LASSO-selected morphological and clinical features of stroma. Mor.: Morphological features, AR: All Region, SR: Significant Regions, LR: Largest Region.. Viewing in color is recommended.

References

1. Janowczyk A, Madabhushi A: **Deep learning for digital pathology image analysis: A comprehensive tutorial with selected use cases**. *Journal of pathology informatics* 2016, **7**:29-29.

2. Chollet F: **Xception: Deep learning with depthwise separable convolutions**. In: *Proceedings of the IEEE conference on computer vision and pattern recognition: 2017*; 2017: 1251-1258.

3. Saltz J, Gupta R, Hou L, Kurc T, Singh P, Vu N, Samaras D, Shroyer KR, Zhao T, Batiste R *et al*: **Spatial Organization and Molecular Correlation of Tumor-Infiltrating Lymphocytes Using Deep Learning on Pathology Images**. *Cell Reports* 2018, **23**(1):181-+.

4. Dagher J, Delahunt B, Rioux‐Leclercq N, Egevad L, Coughlin G, Dunglison N, Gianduzzo T, Kua B, Malone G, Martin B: **Assessment of tumour‐associated necrosis provides prognostic information additional to World Health Organization/International Society of Urological Pathology grading for clear cell renal cell carcinoma**. *Histopathology* 2019, **74**(2):284-290.

5. Simonyan K, Zisserman A: **Very deep convolutional networks for large-scale image recognition**. *arXiv preprint arXiv:14091556* 2014.
